# Advancements in Immunotherapy for Breast Cancer: Mechanisms, Efficacy, and Future Directions

**DOI:** 10.7759/cureus.68351

**Published:** 2024-08-31

**Authors:** Archita Rai, Swati G Deshpande, Ashish Vaidya, Raju K Shinde

**Affiliations:** 1 General Surgery, Jawaharlal Nehru Medical College, Datta Meghe Institute of Higher Education and Research, Wardha, IND; 2 Oncology, Jawaharlal Nehru Medical College, Datta Meghe Institute of Higher Education and Research, Wardha, IND

**Keywords:** adoptive cell therapy, cancer vaccines, monoclonal antibodies, checkpoint inhibitors, immunotherapy, breast cancer

## Abstract

Breast cancer is a major global health challenge characterized by its diverse biological behavior and varying treatment responses. Traditional therapies, including surgery, radiation, chemotherapy, hormonal therapy, and targeted therapy, have significantly advanced breast cancer treatment but are often limited by issues such as resistance, side effects, and variable efficacy. Immunotherapy has emerged as a transformative approach, leveraging the body's immune system to target and eliminate cancer cells. This review provides a comprehensive overview of recent advancements in immunotherapy for breast cancer, detailing the mechanisms of various therapeutic strategies, including checkpoint inhibitors, monoclonal antibodies, cancer vaccines, adoptive cell therapy, and oncolytic virus therapy. We evaluate the efficacy of these approaches in different stages of breast cancer, highlighting successes and challenges encountered in clinical settings. The review also addresses the current limitations of immunotherapy, such as treatment-related adverse effects, resistance mechanisms, and issues of cost and accessibility. We discuss promising future directions, including emerging targets, combination therapies, and personalized medicine approaches. By integrating recent research and clinical trial data, this review aims to elucidate the potential of immunotherapy to revolutionize breast cancer treatment, offering insights into its future role in improving patient outcomes and shaping the landscape of oncological care.

## Introduction and background

Breast cancer is one of the most common cancers affecting women worldwide and represents a major health challenge due to its diverse biological behavior and clinical manifestations [1]. This malignancy is primarily categorized into several types, each with distinct characteristics and treatment implications. The most prevalent form is invasive ductal carcinoma (IDC), which originates in the milk ducts and invades surrounding tissues [2]. Invasive lobular carcinoma (ILC), another significant type, starts in the lobules where milk is produced and often presents with a unique growth pattern that can complicate detection. Triple-negative breast cancer (TNBC) is notable for its lack of hormone receptor expression and HER2 overexpression, making it particularly aggressive and challenging to treat. HER2-positive breast cancer, characterized by the overexpression of the HER2 protein, also presents specific treatment challenges but has seen significant advancements due to targeted therapies [3].

Breast cancer staging is a critical component of diagnosis and treatment planning, ranging from stage 0 (carcinoma in situ) to stage IV (distant metastases). The staging process involves assessing tumor size, lymph node involvement, and the presence of distant spread, which collectively inform prognosis and treatment strategies [4]. Standard treatments for breast cancer have evolved significantly and include surgery, radiation therapy, chemotherapy, hormonal therapy, and targeted therapy. Surgical options such as lumpectomy or mastectomy are chosen based on tumor characteristics and patient preferences [5]. Radiation therapy is frequently used post-surgery to minimize the risk of recurrence, while chemotherapy is employed to address both localized and metastatic disease. Hormonal therapies target hormone receptor-positive cancers, and targeted therapies like trastuzumab are used for HER2-positive cancers. Despite these advancements, current treatments are not without limitations, including issues of resistance, adverse side effects, and varying effectiveness across different patient populations [5].

Immunotherapy represents a paradigm shift in cancer treatment, focusing on leveraging the body’s immune system to recognize and combat cancer cells. Unlike traditional therapies that directly target cancer cells, immunotherapy aims to enhance or modify the immune system’s natural ability to identify and destroy malignant cells [6]. This approach encompasses several mechanisms, including the activation of immune cells such as T cells and natural killer cells, inhibition of immune checkpoints that otherwise suppress the immune response, development of cancer vaccines to educate the immune system, and adoptive cell transfer where a patient’s own immune cells are engineered to target cancer more effectively [6]. The development of immunotherapy in oncology has seen remarkable progress over recent decades. Initial efforts were centered around non-specific immune stimulation using cytokines, which showed limited success [7]. The field advanced with the introduction of monoclonal antibodies in the 1980s and 1990s, which allowed for more precise targeting of cancer-related antigens. The real breakthrough came with the advent of checkpoint inhibitors, such as ipilimumab and pembrolizumab, which significantly improved outcomes across various cancers by overcoming immune evasion mechanisms employed by tumors [7]. The purpose of this review is to thoroughly examine the advancements in immunotherapy for breast cancer, focusing on the mechanisms driving these therapies, assessing their efficacy, and exploring future directions. By synthesizing recent research and developments, this review aims to elucidate the potential of immunotherapy to revolutionize breast cancer treatment, offering new hope for improved patient outcomes and novel therapeutic strategies.

## Review

Mechanisms of immunotherapy in breast cancer

Checkpoint Inhibitors

Checkpoint inhibitors (ICIs) have emerged as a groundbreaking approach in the treatment of breast cancer, particularly for subtypes like triple-negative breast cancer (TNBC). These therapies target specific proteins that regulate immune responses, enhancing the body's ability to recognize and destroy cancer cells. The primary mechanisms of action involve inhibiting the PD-1/PD-L1 and CTLA-4 pathways [8]. Programmed cell death protein 1 (PD-1) is a checkpoint protein found on T cells that inhibits T cell activation when engaged by its ligands (PD-L1 and PD-L2). This interaction allows cancer cells to evade immune detection. By blocking PD-1, inhibitors can reactivate T cells, allowing them to attack cancer cells effectively. PD-L1, often overexpressed on various cancer cells, including some breast cancer cells, similarly prevents T cell activation. Inhibitors targeting PD-L1, therefore, disrupt this mechanism of immune evasion [9]. Conversely, cytotoxic T lymphocyte antigen 4 (CTLA-4) downregulates immune responses by competing with the co-stimulatory receptor CD28 for binding to CD80/CD86 on antigen-presenting cells. Inhibiting CTLA-4 enhances T cell activation and proliferation, further bolstering anti-tumor immunity [10].

Several specific checkpoint inhibitors are currently in use or under investigation for breast cancer treatment. Pembrolizumab (Keytruda), a PD-1 inhibitor, has been approved for combination with chemotherapy in patients with advanced TNBC [11]. Nivolumab (Opdivo), another PD-1 inhibitor, is also being explored in clinical trials for various breast cancer subtypes. Atezolizumab (Tecentriq), a PD-L1 inhibitor, has been approved for use in combination with nab-paclitaxel for TNBC, while durvalumab (Imfinzi) and avelumab (Bavencio) are under investigation for their effectiveness in breast cancer. Additionally, ipilimumab (Yervoy), a CTLA-4 inhibitor, is primarily used in combination with PD-1 inhibitors in clinical trials targeting breast cancer [11]. Clinical trials have demonstrated the efficacy of these checkpoint inhibitors, particularly in TNBC. The KEYNOTE-355 trial evaluated pembrolizumab combined with chemotherapy in patients with advanced TNBC, revealing a significant improvement in progression-free survival (PFS) for those with high PD-L1 expression compared to chemotherapy alone [12]. Similarly, the IMpassion031 trial assessed atezolizumab combined with chemotherapy in early-stage TNBC and showed improved PFS and overall survival (OS), leading to its approval for this indication. The CheckMate 142 study investigated the combination of nivolumab and ipilimumab in metastatic breast cancer, yielding promising results, particularly in patients with a high tumor mutational burden [13]. Despite these advancements, challenges remain in the field of immunotherapy for breast cancer. Identifying biomarkers to predict patient responses to checkpoint inhibitors is crucial for optimizing treatment strategies. Additionally, managing immune-related adverse events (irAEs), which can arise from heightened immune activity, is essential for ensuring patient safety and improving treatment tolerability. Future research will focus on refining these therapies and exploring combination strategies to enhance efficacy while minimizing toxicity in breast cancer treatment [14].

Monoclonal Antibodies

Monoclonal antibodies (mAbs) have become a cornerstone of cancer therapy, especially in treating breast cancer. They work through various mechanisms to enhance the immune system's ability to identify and eliminate cancer cells [15]. A key mechanism involves binding to specific proteins on the surface of cancer cells, such as the HER2 receptor in HER2-positive breast cancer. For example, trastuzumab (Herceptin) targets HER2, inhibiting cell proliferation and promoting apoptosis in cancer cells. Additionally, mAbs can mediate antibody-dependent cellular cytotoxicity (ADCC), a process where immune cells are recruited to destroy cancer cells that the antibodies have marked. They can also activate the complement system, leading to the lysis of cancer cells, and block growth signals, further inhibiting tumor progression [16]. Recent advancements in monoclonal antibody therapy have expanded the range of targets beyond traditional markers like HER2. One notable development is the creation of bispecific antibodies, engineered to bind to two different antigens simultaneously, thereby enhancing the immune response [17]. For instance, bispecific T cell engagers (BiTEs) can direct T cells more effectively to cancer cells. Moreover, immune checkpoint inhibitors, which are mAbs targeting proteins such as PD-1/PD-L1 and CTLA-4, are being integrated into treatment regimens. These therapies boost the immune system's ability to recognize and attack tumors. Another promising innovation is the development of antibody-drug conjugates (ADCs), which combine mAbs with cytotoxic drugs. This allows for targeted delivery of chemotherapy directly to cancer cells while minimizing systemic toxicity [18].

Regarding clinical efficacy, monoclonal antibodies have demonstrated significant benefits across various breast cancer subtypes. In HER2-positive breast cancer, trastuzumab has been shown to improve overall survival and reduce recurrence rates [19]. The addition of pertuzumab, another HER2-targeted mAb, has further enhanced treatment efficacy, leading to better patient outcomes. In triple-negative breast cancer (TNBC), recent studies have explored the use of mAbs in combination with chemotherapy and immune checkpoint inhibitors, showing promising results in improving response rates and survival [19]. While mAbs generally have a favorable safety profile, they can be associated with specific adverse effects. Common side effects include infusion reactions, an increased risk of infections, and potential cardiac toxicity, particularly with HER2-targeted therapies. Therefore, monitoring and management strategies are essential to mitigate these risks during treatment [20].

Cancer Vaccines

Cancer vaccines represent a promising area of immunotherapy aimed at treating existing cancer vaccines, designed to stimulate the immune system to target and destroy cancer cells or prevent their recurrence. These vaccines can be classified into several types, each with distinct mechanisms of action. Protein or peptide vaccines consist of specific proteins or peptides found in cancer cells, which activate the immune system to recognize and attack these cells [21]. DNA and RNA vaccines use genetic material encoding cancer-specific antigens, prompting the body's cells to produce these antigens and thereby enhancing the immune response. Whole-cell vaccines use entire cancer cells, which may be modified to improve immune recognition. In contrast, dendritic cell vaccines involve harvesting dendritic cells from the patient, exposing them to cancer antigens, and reintroducing them to stimulate a strong immune response. Additionally, viral vector vaccines employ modified viruses to deliver cancer antigens into the body, effectively stimulating an immune response [22]. The development of cancer vaccines focuses on training the immune system to recognize and attack cancer cells. This process is key to identifying tumor-associated antigens (TAAs) and tumor-specific antigens (TSAs). TAAs are proteins overexpressed on cancer cells compared to normal cells, while TSAs result from mutations unique to cancer cells [23]. To boost the immune response, many vaccines incorporate adjuvants, which enhance the vaccine's effectiveness. Furthermore, combining cancer vaccines with other treatments, such as checkpoint inhibitors or chemotherapy, is an emerging strategy to improve overall efficacy by overcoming the immune suppression often associated with tumors [24]. The landscape of cancer vaccines is characterized by extensive research and numerous clinical trials. Approximately 360 active clinical trials are investigating various types of cancer vaccines, exploring different delivery methods, antigen types, and cancer indications. These trials cover a wide range of cancers, including breast cancer, lung cancer, and melanoma [25]. While only a few cancer vaccines have received regulatory approval, notable examples include sipuleucel-T for prostate cancer and Bacillus Calmette-Guérin (BCG) for bladder cancer. Talimogene laherparepvec, an oncolytic virus vaccine, is also approved for the treatment of melanoma. Despite their potential, many cancer vaccines have not yet achieved significant clinical success due to challenges such as identifying suitable antigens, personalized approaches, and the requirement for effective immune responses in patients whose immune systems may be compromised by cancer [26].

Adoptive Cell Therapy

Adoptive cell therapy (ACT) is an innovative cancer treatment approach that harnesses the body's immune cells to target and eliminate cancer cells. This therapy includes several modalities, each with distinct mechanisms of action and targeting strategies [27]. The primary forms of ACT are chimeric antigen receptor T cell (CAR-T) therapy, tumor-infiltrating lymphocyte (TIL) therapy, T cell receptor (TCR) therapy, natural killer (NK) cell therapy, and macrophage-based therapies. CAR-T therapy involves genetically engineering a patient's T cells to express a chimeric antigen receptor that targets specific cancer cell antigens. This method has demonstrated remarkable success in treating hematologic malignancies, such as acute lymphoblastic leukemia and certain types of lymphoma [28]. Conversely, TIL therapy utilizes immune cells that have naturally infiltrated a tumor. These lymphocytes are extracted, expanded in the laboratory, and then reinfused into the patient. TIL therapy has proven effective in treating melanoma and is currently being investigated for use in other solid tumors [29]. The underlying mechanisms of these therapies focus on enhancing the immune system's ability to identify and destroy cancer cells. For example, CAR-T cells are engineered to bind to specific antigens on tumor cells, marking them for destruction. Upon recognizing cancer cells, these engineered T cells or NK cells activate, proliferate, and release cytotoxic molecules that induce cancer cell death [30]. Additionally, many forms of ACT use cytokines to support T cell survival and boost their anti-tumor activity. TIL therapy, for instance, often involves the administration of interleukin-2 (IL-2) to promote T cell expansion and function. A significant challenge to the effectiveness of ACTs is the tumor microenvironment (TME), which can suppress immune responses. Researchers are exploring strategies to modify the TME to enhance the efficacy of adoptive cell therapies, such as combining these therapies with immune checkpoint inhibitors [31]. Current research in ACT is rapidly advancing, with numerous clinical trials underway. As of 2021, five FDA-approved CAR-T therapies are available for various blood cancers, and ongoing trials are exploring their use in solid tumors [32]. Researchers are focused on improving CAR designs to increase efficacy and minimize side effects. TIL therapy is also showing promise in clinical trials for advanced melanoma and cervical cancer, with some therapies receiving breakthrough designations [33]. These trials are investigating optimal conditions for TIL expansion and reinfusion. While TCR therapies are still largely experimental, they are being tested in multiple cancers, and early results suggest potential effectiveness against tumors with specific antigen profiles. Additionally, CAR-NK cell therapies are in early clinical trials and show promise for treating hematologic malignancies and solid tumors. Research in this area concentrates on improving NK cells' persistence and efficacy within the TME [34].

Oncolytic Virus Therapy

Oncolytic virus therapy represents a novel and promising approach to cancer treatment, utilizing genetically modified viruses to selectively target and destroy cancer cells while stimulating the immune system. This therapeutic strategy functions through two primary mechanisms [35]. First, oncolytic viruses (OVs) directly infect and replicate within cancer cells, leading to cell lysis without harming normal cells. This selective replication is possible because cancer cells often have impaired antiviral defenses, providing an optimal viral replication environment and ultimately leading to their destruction [35]. In addition to direct oncolysis, oncolytic viruses stimulate anti-tumor immunity. The destruction of cancer cells releases tumor-associated antigens, which can trigger a systemic immune response. This process enhances the recruitment and activation of immune cells, enabling a broader attack on remaining tumor cells. This approach can also generate immunological memory, potentially offering long-term protection against cancer recurrence [36]. Several oncolytic viruses are currently being explored in clinical trials for breast cancer treatment. A notable example is talimogene laherparepvec (T-VEC), a genetically modified herpes simplex virus type 1 (HSV-1). While T-VEC has shown significant promise in treating melanoma, it is now being investigated for its efficacy in breast cancer [37]. T-VEC is engineered to enhance local immune responses while directly lysing tumor cells. Other oncolytic viruses under investigation include modified adenoviruses and vaccinia viruses, both of which have been studied for their capacity to selectively infect and kill breast cancer cells while promoting an immune response. These viruses can be genetically engineered to express therapeutic genes that enhance their oncolytic properties [38].

**Figure 1:**
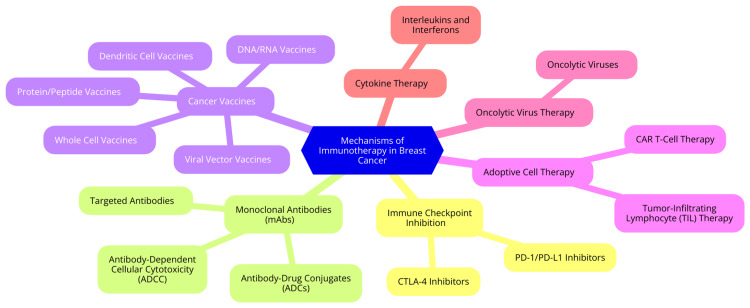
Mechanisms of immunotherapy in breast cancer Image Credit: Dr Archita Rai CAR - chimeric antigen receptor

Clinical trials involving oncolytic viruses have shown varying levels of success. Initial studies have demonstrated that these therapies can result in tumor regression and improved immune responses in breast cancer patients [39]. For example, trials with T-VEC have indicated enhanced local tumor control and systemic immune activation. However, outcomes can vary based on the type of tumor and individual patient characteristics. Despite these promising results, challenges persist, including optimizing viral delivery, ensuring patient safety, and managing immune-related side effects. The unique biology of each tumor necessitates customized approaches to maximize therapeutic effectiveness [39]. Ongoing research aims to refine oncolytic virus therapies through genetic modifications that enhance their efficacy and safety. Combining OVs with other treatment modalities, such as immune checkpoint inhibitors or conventional chemotherapy, is also being explored to improve patient outcomes [40]. The potential approval of additional oncolytic viruses by regulatory bodies may accelerate their development and integration into standard cancer treatment protocols. Overall, oncolytic virus therapy is promising in breast cancer treatment by leveraging direct tumor destruction and immune system activation. Continued research and clinical trials will be vital in overcoming current challenges and establishing effective treatment regimens [40]. Mechanisms of immunotherapy in breast cancer are shown in Figure 1.

Efficacy of immunotherapy in breast cancer

Immunotherapy has demonstrated promising results in the treatment of early-stage breast cancer, especially when combined with chemotherapy. For example, the KEYNOTE-522 trial showed that neoadjuvant pembrolizumab, an immune checkpoint inhibitor (ICI), significantly increased pathologic complete response (pCR) rates in patients with early-stage triple-negative breast cancer (TNBC) compared to chemotherapy alone. In this trial, pCR rates were approximately 64.8% in the pembrolizumab group versus 51.2% in the chemotherapy-only group. These findings suggest that integrating immunotherapy into the treatment regimen for early-stage breast cancer can lead to improved tumor response rates, making it a valuable addition to conventional treatments [41]. Beyond higher pCR rates, the integration of ICIs in early treatment settings has been linked to enhanced event-free survival. The positive outcomes observed in clinical trials underscore the potential of immunotherapy to alter the disease course in early-stage breast cancer, providing a strong rationale for its use in combination with traditional therapies. This combination approach could lead to better long-term outcomes for patients, highlighting the evolving role of immunotherapy in early breast cancer management [42]. In advanced and metastatic breast cancer, particularly TNBC, ICIs have also shown significant efficacy. The KEYNOTE-355 trial demonstrated that pembrolizumab combined with chemotherapy improved progression-free survival (PFS) and overall survival (OS) in patients with high PD-L1 expression. Specifically, patients with a combined positive score (CPS) greater than 10 experienced significant benefits from this combination therapy. These results highlight the effectiveness of immunotherapy in a population with historically limited treatment options and poor prognosis, offering new hope for better outcomes in advanced disease settings [43].

When comparing immunotherapy to traditional treatments, ICIs have provided more durable responses in certain subsets of patients. While chemotherapy remains a cornerstone of treatment for advanced breast cancer, the addition of immunotherapy has led to improved outcomes in specific populations, particularly those with aggressive disease characteristics like TNBC. This shift in treatment paradigms underscores the growing importance of immunotherapy in managing advanced breast cancer and its potential to offer sustained clinical benefits where traditional therapies may fall short [44]. Biomarkers play a crucial role in predicting responses to immunotherapy in breast cancer. Key biomarkers include PD-1/PD-L1 expression, tumor mutational burden (TMB), and the presence of tumor-infiltrating lymphocytes (TILs). High levels of TILs, particularly in TNBC, are associated with better prognosis and response to immunotherapy, indicating that the immune landscape of the tumor significantly influences treatment efficacy. Understanding these biomarkers is essential for optimizing patient selection for immunotherapy, ensuring that patients most likely to benefit from these therapies are identified and treated appropriately [45]. The identification and use of biomarkers allow for more personalized treatment strategies for breast cancer. By tailoring immunotherapy based on individual tumor characteristics, clinicians can optimize treatment plans and improve patient outcomes. Ongoing research aims to refine these predictive models, enhancing the ability to select patients most likely to benefit from immunotherapy. This personalized approach not only improves efficacy but also minimizes unnecessary exposure to ineffective treatments, thereby reducing potential side effects and improving overall quality of life for patients [46].

Challenges and limitations

Immunotherapy has revolutionized the treatment landscape for breast cancer, providing new hope, particularly for aggressive subtypes like triple-negative breast cancer (TNBC). However, this innovative approach is not without its challenges and limitations. Understanding these issues is crucial for optimizing patient care and improving outcomes [47]. One of the primary challenges associated with immunotherapy is the occurrence of treatment-related adverse effects. While many patients experience common side effects such as fatigue, rash, diarrhea, and nausea, severe immune-related adverse events (irAEs) can also occur, affecting various organs. These severe effects may include endocrine dysfunctions like thyroiditis, gastrointestinal issues such as colitis, pulmonary complications like pneumonitis, and liver-related problems such as hepatitis [48]. Effective management of these adverse effects is essential for maintaining patient quality of life and ensuring treatment adherence. Key strategies for mitigating these challenges include regular monitoring of patients for early detection of irAEs, symptomatic treatments (such as corticosteroids for severe reactions), patient education about potential side effects, and dose adjustments based on the severity of adverse effects [49]. Another significant concern is the development of resistance to immunotherapy. Resistance can arise from various factors, including the tumor microenvironment, genetic alterations, upregulation of immune checkpoints, and T cell exhaustion. An immunosuppressive tumor microenvironment can inhibit T cell activation and function, while genetic mutations may prevent effective immune recognition of cancer cells [50].

Additionally, tumors may exploit alternative immune checkpoints to evade immune responses. Researchers are exploring combination therapies that use immune checkpoint inhibitors alongside other treatments, such as chemotherapy or targeted therapies, to overcome these resistance mechanisms. Furthermore, targeting the tumor microenvironment with agents that modify its immunosuppressive properties and personalizing immunotherapy based on individual tumor characteristics are promising strategies that may enhance treatment efficacy [50]. Economic considerations and healthcare access issues also pose significant challenges to the widespread adoption of immunotherapy. The high cost of these treatments, which can exceed tens of thousands of dollars annually, creates barriers for many patients. Insurance coverage variation can further limit access, particularly for those without comprehensive health plans. Socioeconomic factors can lead to disparities in care, adversely affecting patient outcomes [51]. The financial burden associated with immunotherapy may lead to treatment abandonment or delays, negatively impacting prognosis. Additionally, financial concerns can complicate treatment adherence and overall well-being. Addressing these economic barriers is essential to ensure equitable access to immunotherapy for all patients, regardless of their financial situation [52].

Future directions in immunotherapy for breast cancer

Emerging Targets and Technologies

Research increasingly identifies new molecular targets for immunotherapy in breast cancer, expanding the potential for more effective treatments. Notable developments include chimeric antigen receptor (CAR) T cell therapy, a cutting-edge approach involving the genetic engineering of T cells to express CARs that target specific breast cancer antigens. This therapy is particularly promising for aggressive subtypes like triple-negative breast cancer (TNBC). Ongoing studies are exploring CAR T cells targeting antigens such as HER2 and ROR1, which are relevant in TNBC and other difficult-to-treat breast cancer subtypes. Additionally, bispecific antibodies, which can bind simultaneously to two different antigens, are being investigated to enhance the immune response against cancer cells while minimizing effects on healthy tissues. This dual-targeting capability aims to increase the precision and effectiveness of immunotherapy while reducing the risk of off-target effects [53]. Technological innovations are playing a crucial role in advancing the field of immunotherapy for breast cancer. Next-generation sequencing (NGS) has become a vital tool, enabling the identification of neoantigens-mutated peptides unique to cancer cells and absent in normal cells. This capability facilitates the development of personalized vaccines and T cell therapies tailored to the specific genetic mutations in an individual's cancer, allowing for highly targeted immune responses. Moreover, artificial intelligence (AI) is being increasingly utilized to analyze complex datasets derived from genomic studies. AI technologies can help predict patient responses to immunotherapy, optimize treatment strategies, and identify new molecular targets for potential therapeutic intervention. By integrating these technological advancements, researchers enhance our understanding of breast cancer biology and pave the way for more precise and personalized immunotherapy approaches [54].

Combination Therapies

Combining immunotherapy with traditional treatments, such as chemotherapy and targeted therapies, is an emerging strategy that holds considerable promise for enhancing the effectiveness of cancer treatment. Evidence suggests that immune checkpoint inhibitors (ICIs), when combined with chemotherapy, have demonstrated improved outcomes in patients with triple-negative breast cancer (TNBC). This has led to the FDA approving several combinations of ICIs and chemotherapy for treating this aggressive subtype. The rationale behind these combinations is that chemotherapy can potentially enhance the effectiveness of ICIs by increasing the release of tumor antigens and reducing immunosuppressive cells in the tumor microenvironment, thereby making cancer more susceptible to immune attack. Additionally, the combination of CAR T cell therapy with ICIs is under investigation to further enhance the efficacy of both treatments. The potential of this combination lies in its ability to generate sustained and robust immune responses against tumors, potentially overcoming some of the resistance mechanisms that limit the effectiveness of either therapy alone. By targeting different aspects of the immune response and tumor biology, combination therapies aim to improve long-term outcomes for patients with difficult-to-treat cancers like TNBC [55]. The rationale for combination therapies is their ability to address tumor heterogeneity and counteract resistance mechanisms. Tumors often comprise a diverse mix of cancer cells with different genetic and molecular characteristics, making it challenging for a single treatment modality to be universally effective. Combining different therapeutic approaches, such as chemotherapy, targeted therapy, and immunotherapy, it can attack the tumor on multiple fronts, targeting various pathways that cancer cells may exploit to evade the immune system. Studies indicate that such combinations can produce synergistic effects, enhancing overall treatment efficacy and improving patient outcomes. This comprehensive approach allows for a more effective attack on the tumor, potentially leading to better patient survival rates and quality of life [56].

Personalized and Precision Medicine

Personalized medicine is increasingly becoming a cornerstone of breast cancer treatment. Advances in neoantigen-based vaccines, customized based on individual tumor profiles, offer the potential for more effective immune responses. Additionally, ongoing research aims to identify biomarkers that predict patient responses to specific immunotherapies, enabling more tailored treatment strategies. This emphasis on individualization is vital for ensuring patients receive the most effective therapies suited to their unique cancer characteristics [57]. Future research will likely focus on refining the identification of effective targets and biomarkers and developing more sophisticated models to predict patient responses to immunotherapy. This may include integrating multi-omics approaches to gain a deeper understanding of tumor biology and the immune landscape, ultimately leading to breakthroughs in treatment strategies [58].

Global Perspectives and Trials

Numerous clinical trials are currently underway worldwide, investigating various immunotherapeutic strategies for breast cancer. These trials include assessments of immune checkpoint inhibitors (ICIs) in combination with other therapies, exploring their efficacy across different breast cancer subtypes. Additionally, global studies are evaluating the effectiveness of adoptive cell therapies, such as tumor-infiltrating lymphocyte (TIL) therapy and CAR T cell therapy, particularly in the advanced stages of the disease [59]. International collaboration is crucial for advancing breast cancer immunotherapy. Sharing data and insights from diverse populations can enhance the understanding of treatment responses and facilitate the development of more effective therapies. Collaborative networks enable multi-center trials, which accelerate the pace of research and innovation in this field. By pooling their collective expertise, researchers and clinicians can improve outcomes for breast cancer patients worldwide [60].

## Conclusions

In conclusion, the advancements in immunotherapy represent a significant breakthrough in the treatment of breast cancer, offering new avenues for managing this complex and heterogeneous disease. The development of checkpoint inhibitors, monoclonal antibodies, cancer vaccines, adoptive cell therapies, and oncolytic virus therapies has expanded the arsenal of treatment options, providing new hope for patients, particularly those with aggressive or resistant forms of the disease. Despite the promising efficacy and potential benefits, challenges remain, including treatment-related adverse effects, mechanisms of resistance, and issues related to cost and accessibility. As research continues to evolve, future directions in immunotherapy will likely focus on refining these therapies, exploring novel targets, and integrating them with other treatment modalities to enhance overall efficacy. The goal is to move towards more personalized and precise treatment strategies that address the unique characteristics of each patient's cancer, ultimately improving outcomes and quality of life. As the field progresses, it is imperative to address these challenges while continuing to advance our understanding and application of immunotherapy in breast cancer, paving the way for transformative changes in patient care and treatment paradigms.
